# Emergence of a new me: Experiences of women diagnosed with HER2-positive breast cancer

**DOI:** 10.1371/journal.pone.0325080

**Published:** 2025-07-10

**Authors:** Josianne Scerri, Janice Agius, Michael Galea

**Affiliations:** 1 Department of Mental Health, Faculty of Health Sciences, University of Malta, Msida, Malta; 2 Faculty of Health, Science, Social Care and Education, Kingston University, Surrey, United Kingdom; Local Health Authority Caserta: Azienda Sanitaria Locale Caserta, ITALY

## Abstract

Breast cancer subtypes vary in prevalence, prognosis, treatment protocols, and side-effect profiles, that may impact patients’ lived experiences. Despite this, studies often group the experiences of persons with breast cancer together. The aim of this study was to explore the lived experiences of non-metastatic women diagnosed with Herceptin2-positive breast cancer, from diagnosis to the completion of treatment. Semi-structured interviews were conducted with 13 women who had undergone treatment for HER2 + breast cancer within the past two years. Their ages ranged between 32–79 years. Data collected were analysed using interpretative phenomenological analysis. Four experiential themes emerged: ‘My world came crashing down,’ ‘The countdown,’ ‘To do or not to do, that is the question,’ and ‘Nearing the end of my journey.’ These themes correspond to four phases of the participants’ experiences: (i) the diagnostic phase, (ii) the neoadjuvant chemotherapy phase, (iii) the surgical phase, and (iv) the post-surgery adjuvant phase. The diagnosis elicited strong emotional reactions due the particularly aggressive nature of this type of cancer. However, the availability of targeted treatment and having a relatively common subtype of breast cancer provided hope for recovery. Throughout the neoadjuvant phase, participants experienced a range of emotions, including anxiety over missing any treatment, distress about changes in body-image, and concerns for their families. As treatment approached its end, they felt relieved but also anxious about the possibility of a recurrence. Understanding the narratives of women having Herceptin2-positive breast cancer, allows health professionals to better address their unique needs, concerns, and values, leading to the development of personalised care plans that target their lived experiences.

## Introduction

Breast cancer remains one of the most prevalent cancers worldwide, predominantly affecting women [1]. In 2020, over 2.26 million women received a breast cancer diagnosis, constituting approximately 25.8% of all female cancer cases [2]. The heterogenous nature of breast cancer makes its prognosis, treatment, and severity difficult to foresee. Consequently, the classification of breast cancer into various subtypes, based on standard immunohistochemical markers [3,4], aids in tailoring therapy plans and predicting therapeutic outcomes.

A specific subtype, HER2+ (human epidermal growth factor receptor 2) is characterised by an overexpression of the HER2 oncogene [5] and accounts for 15%−30% of all breast cancers [6,7]. The overexpression and rapid multiplication of HER2 biomarkers results in aggressive cancer growth, leading to poor prognosis, low survival rates and an increased likelihood of recurrence [5,8]. Moreover, HER2 + breast cancer is associated with metastasis, particularly in the lymph nodes and brain [3,4].

Recent advances in the treatment of HER2 + breast cancer have enhanced prognosis and overall survival rates, particularly in its initial stages [9]. Targeted therapy anticancer drugs, notably Trastuzumab, have significantly improved the prognostic landscape and quality of life for HER2 + breast cancer patients [10]. Current guidelines recommend the use of Trastuzumab, and the monoclonal antibody Pertuzumab, combined with chemotherapy as the primary treatment for HER2 + breast cancer [11,12]. This approach aims to shrink the tumour before surgical removal, with the choice between lumpectomy and mastectomy, depending on the tumour’s size and location. Adjuvant post-surgery treatments provided vary based on the presence of any residual cancer cells and typically involve a combination of anti-HER2 therapy for several weeks, or a year-long combination treatment of Trastuzumab and Pertuzumab [13]. Endocrine therapy is additionally recommended for hormone receptor-positive and HER2 + breast cancer to mitigate the risk of treatment resistance [14].

Receiving a breast cancer diagnosis often leads to psychological distress, due to concerns about the disease’s prognosis, treatment process, and associated mortality rates. Various qualitative studies highlight the significant psychological and physical challenges faced by those living with breast cancer [15,16]. These challenges include the shock of receiving the diagnosis, a sense of losing control over their lives, and the burden of chemotherapy side-effects such as altered taste, sexual dysfunction, and difficulty concentrating. The emotional impact experienced is further compounded by changes in body image, the threat of death, and the difficulties in discussing the illness with family and friends [16–18].

While many studies examine the experiences of living with breast cancer, they frequently merge the experiences of individuals with various subtypes together. This approach can overlook the distinct prognoses, treatment protocols, and side-effect profiles that significantly affect quality of life. In contrast, Galipeau et al.’s [19] study specifically targets HER2 + breast cancer but focuses solely on metastatic cases, which typically have a lower quality of life compared to non-metastatic cases [20]. This study aims to fill a gap in the literature by examining the experiences of non-metastatic HER2 + breast cancer patients throughout the diagnostic and treatment phases. The findings generated may assist healthcare professionals in formulating personalized care plans tailored to the needs of HER2 + breast cancer patients.

## Methods

### Study design

The qualitative design selected was Interpretative Phenomenological Analysis (IPA), as it allows for an in-depth exploration of participants’ lived experiences while recognising the researcher’s interpretative role [21]. IPA is grounded in three theoretical foundations: phenomenology, hermeneutics, and ideography. Phenomenology aims to capture participants’ lived experiences through their subjective first-person narratives, whereas hermeneutics focuses on the analyst’s effort to understand how participants make sense of their experiences [22]. The ideographic nature of the present study is reflected in the detailed analysis of each individual case, before conducting cross-comparisons among different cases.

### Participants and setting

The recruitment period for this study was between the 26^th^ of July and 1^st^ of December 2023. Purposive sampling was used to recruit thirteen adults who had undergone treatment for HER2 + breast cancer within the past two years. These participants were all female, non-metastatic and were in remission. Their ages ranged between 32–79 years (mean age: 50.5 years). Of these, 76.9% of participants were in a relationship and had children, 61.5% were employed, 15.4% had retired from work, and 23.1% were homemakers. Table 1 presents the demographic details for study participants.

**Table 1:** Demographic details of participants.

Pseudonym	Age (years)	Employment status	Relationship status- (Yes/No)	Have children (Yes/No)
Lara	67	Retired	Yes	Yes
Stefania	38	Homemaker	Yes	Yes
Maria	59	Employed	No	No
Anne	58	Employed	Yes	Yes
Alexia	58	Employed	No	Yes
Francesca	35	Employed	Yes	Yes
Kate	59	Employed	Yes	Yes
Christine	32	Employed	Yes	Yes
Amy	36	Homemaker	Yes	No
Kristina	36	Homemaker	Yes	Yes
Daniela	79	Retired	No	Yes
Janet	55	Employed	Yes	Yes
Carla	45	Employed	Yes	No

### Data collection

The study consisted of audio-recorded interviews that were conducted by the first author [JS]. Participants were invited to describe their lived experiences following a diagnosis of HER2 + breast cancer and throughout active treatment. Probing questions like ‘how did your treatment regimen impact your quality of life and possibly that of significant others in your life, if in any way?’ were used to gain further insight when required. The duration of the interviews ranged between 40–90 minutes, and they were transcribed verbatim.

### Data analysis

Data analysis adhered to Smith et al.’s [21] guidelines. This involved a careful reading and re-reading of the transcript on a case-by-case basis by authors. Throughout this process, exploratory statements emphasising descriptive, linguistic, and conceptual insights were generated manually for each case. Extracts from the data were coded and experiential statements were formulated by the first author [JS]. Those statements with similar understandings were grouped into personal experiential themes for each case. A group experiential statement (GET) was then developed by exploring individual cases for patterns of convergence and divergence. The authors then discussed the GETs generated and came to a final agreement. The process of data collection continued until saturation of the main themes was achieved.

### Ethical considerations

The study was conducted in accordance with the Declaration of Helsinki and approved by the Faculty of Health Sciences research ethics committee, University of Malta [Approval number: FHS2022−00338]. Potential participants received an information sheet through an intermediary (health professional) outlining the nature of the study. Those persons who expressed a willingness to participate, were subsequently contacted by the first author. A meeting was held with each participant to address any queries and provide any clarifications before obtaining their consent. Voluntary participation in the research was obtained through written consent, whilst confidentiality was safeguarded by replacing participant names with pseudonyms. Psychological support was available should any participant experience distress, however this was not availed of.

### Rigor

Yardley’s model [23] was employed to assess the trustworthiness of the study. This model encompasses four dimensions: sensitivity to context, commitment and rigor, coherence and transparency, and impact and importance. Sensitivity to context was achieved through the incorporation of relevant literature and the inclusion of verbatim extracts. Commitment and rigor were ensured by providing comprehensive details on research design, participant recruitment, and data analysis. Coherence and transparency were enabled by providing a rationale for decisions taken and by aligning the study with the underlying theoretical assumptions of IPA. The additional use of a reflexive diary helped identify biases and observations, thereby enhancing transparency. The principle of impact and importance was addressed by delving into an underexplored subject, namely the experiences of adults diagnosed with non-metastatic HER2 + breast cancer.

## Results

Data analysis revealed 4 GETs namely ‘My world came crashing down’, ‘The countdown’; ‘To do or not to do, that is the question’ and ‘Nearing the end of my journey’. These experiential themes reflect the participants’ experiences during the diagnostic phase, and during the following active phases of treatment namely: the neoadjuvant phase (prior to surgery); the surgical phase, and the adjuvant phase (following surgery). Table 2 illustrates the resultant Group experiential themes and subthemes derived from the data collected.

**Table 2:** Group experiential themes and subthemes.

Group experiential Theme	Subthemes	Phase
My world came crashing down	Physically overwhelmed Frequent hospital visits Burdened with emotions Searching for information	Diagnosis
The countdown	Navigating emotions A changed identity Support from family and friends	Neo-adjuvant (i.e., targeted therapies prior to surgery)
To do or not to do…that is the question!	Mixed views about surgery Preparing for surgery	Surgical
Nearing the end of my journey	Destroying any remaining cells Physically less demanding Anxious about future	Adjuvant (i.e., receiving treatment post-surgery)

### My world came crashing down

Receiving a formal diagnosis was a deeply impactful and devastating experience for most participants. Although they had contemplated the possibility of cancer after finding a lump in their breast, the confirmation of the diagnosis caused their “world to come crashing down” (Daniela). In the following excerpt, Amy describes the sense of panic that engulfed her.

“…*there’s a difference when you kind of know that it’s something bad and then, when you confirm it is bad, …it’s totally a different feeling… I began hyperventilating and spent an hour trying to get my breath back*” (Amy)

Another participant vividly compared this moment to a bomb explosion, evoking images of chaos and havoc. Carla described being swept off her feet, attending numerous

medical appointments and procedures within a confined timeframe.

“*You’re living a normal life and then suddenly a bomb explodes just like that and then it’s just appointments, scans, errands… till that time you have never been admitted to hospital, then in two weeks you get to know every nook and cranny that exists*” (Carla)

Two participants however experienced a need to exert some control over the situation. They actively sought out diverse professionals whom they could turn to for advice.

*“Ok what to do? What am I going to do? What am I going to do? Next, next, next, action, action, action…I’m a doer, I try to nip it [a challenge] in the bud...I just wanted to stay in control”* (Maria)

Though several participants were unable to identify their specific subtype of breast cancer, all were aware that they had a particularly aggressive form of the disease. The tumour’s size was an element that intensified such awareness.

*“When he [the surgeon] informed me of its size, I knew immediately it was aggressive… and I knew from its size that it had developed quite a while back, I had never realised before”* (Christine).

This awareness triggered fear and anxiety in them, as they worried about the potential spread of the cancer, especially since they had not started any treatment yet. Additionally, three participants questioned why they were not offered surgery first, rather than neoadjuvant chemotherapy that serves to shrink the tumour.

“…*he [the doctor] told me it’s aggressive and fast spreading, those were his words… I got afraid, … I started thinking that it will spread to other organs whilst I wait for my treatment to commence…I still cannot understand why I didn’t undergo surgery first to get it [cancer] out”* (Kate).

Exposure to individuals who have either “successfully beat it [cancer] or live with it” (as Maria described), along with the knowledge that they have a relatively common subtype of breast cancer with targeted therapies, offered participants comfort and hope for recovery.

*“There’s a lot of research on it [HER2+ breast cancer] and that made me a bit confident as well, because this is a common type of cancer, like the oestrogen-driven cancer, and one of the breast cancers that are most healed, so in a way, that puts my mind somewhat at rest”* (Janet)

Yet for others researching about their type of breast cancer proved disheartening. They described being exposed to the worse case scenarios and being inundated with information that distressed them.

*“I searched; I searched a lot on the internet. I read a lot, but it was making me go crazy. It was stressing me, and I couldn’t sleep, one site says this, another site says that. I then decided that I am not going to search about cancer anymore”* (Alexia)

The participants also expressed significant concerns for their family’s well-being, particularly that of their children. Many struggled with feelings of guilt, believing that their loved ones did not deserve such hardships. Despite these challenges, they showed strong determination to overcome cancer, driven by their desire to witness important milestones in their children’s lives and to be a positive model for them.

*“My children were still young, and I was praying to God that I don’t die, otherwise who would take care of them? I wanted to survive for my kids”* (Christine)

Most mothers refrained from using the word “cancer” with young children. Instead, they prepared their children for possible changes in their appearance and/or behaviour.

*“… I told them Mummy is sick, she has something very serious… I explained that I am not sure if I will get better, I told them there will be physical changes, I might lose my hair, I might be very tired, they might come from school and find me in bed, without energy to help them… in a way I prepared them for the worst”* (Christine)

As participants processed their diagnosis and prepared to begin treatment, they transitioned from a state of shock to proactive engagement, developing a sense of control and hope. They expressed a strong desire to start treatment promptly, seeing it as a crucial step in managing their aggressive cancer and aiming to restore a sense of normalcy to their lives.

*“I focused on wanting the best, so that I can once more be… the mother I was, the woman I was and the daughter I was. Those three.”* (Christine)

### The countdown

This section details the participants’ experiences with chemotherapy and antibody treatment prior to surgery. They navigated a wide range of emotions, including fear of the unknown, concerns about the treatment’s effectiveness, to anxiety about missing any scheduled chemotherapy sessions. Attending these sessions was seen as crucial to avoid extending the duration of the treatment and to reduce the risk of the cancer spreading.

*“My target was to not miss any treatment session. If they told me, it’s every three weeks, I wanted to go every three weeks, because when they told me the duration was of one year and a half, I thought it was too long. So, I started counting down the days until the last planned treatment. But if you miss any treatment, it will be even longer, and the cancer could spread further.”* (Carla)

Avoiding interruptions in treatment was viewed as crucial for controlling an aggressive cancer. Consequently, some participants chose to limit interactions with others to minimise their own risk of falling ill.

*“...My young daughter works in a community setting, which means that she is in constant contact with others. We felt that the best solution was for her to go and live with my sister and that we [the patient and her spouse] stay on our own”* (Stefania)

The loss of hair and/or eyelashes caused significant emotional distress for participants. They believed that hair on the head was essential for a woman to feel complete, and the absence of eyebrows left their face devoid of expression. These changes resulted in a person who was unrecognisable to them.

*“Having hair, being a woman, makes me feel complete, I feel smart, but when you look in the mirror, I say this disease has taken away all my dignity. Even the loss of my eyebrows... without them the face is devoid of expression. When I’d wash and then look into the mirror, I’d say is that me, the same person that I was”* (Carla)

Participants further expressed worry and guilt that they could have genetically transmitted the gene for this aggressive cancer to their children. They also expressed their fear of having to go through a similar experience, if one of their children were to be diagnosed with breast cancer in the future.

*“I am afraid that we will go through the same experience in a few years’ time. That would be even worse because my children would have it… will it be my fault? Had I known I would not have chosen to bring any [children] into the world.”* (Francesca)

Some participants also expressed feelings of anger and bitterness, questioning why they had to go through this experience despite leading a healthy lifestyle.

*“It is not because it happened to me, but when you look at others, they do all sorts of operations to enhance here and there, and they eat junk food. You get angry over this as I really lived a healthy lifestyle.”* (Carla)

Although the participants strove to maintain a sense of normalcy and adhered to their usual routines, they occasionally required support mainly from family and friends. However, this support came with its own consequences. Participants were aware of the emotional turmoil that their diagnosis was causing to loved ones and how they coped with it behind closed doors.

*“… it’s hard also on the person who is supporting. First and foremost, they can’t, like, when I have a breakdown and cry, they support me…they need to be strong for me, they must repress and hide their own feelings and… you know, he [the spouse] just goes and cries when I’m asleep”* (Amy)

### To do or not to do…that is the question!

When deciding on whether to undergo a lumpectomy or a mastectomy, the participants expressed mixed views. Some felt relieved at the possibility of a lumpectomy because it would preserve more of their breast tissue. In contrast, others viewed undergoing a lumpectomy as equivalent to a death sentence. For example, Maria was in favour of a mastectomy, perceiving it as a way to reduce the risk of recurrence.

*“… I told my surgeon I want them both off! It’s [the breasts] a killer for me.”* (Maria)

However, for most participants, undergoing a mastectomy was distressing because they associated their breasts with their feminine identity. As a result, many opted for immediate reconstruction after a mastectomy to avoid the prospect of living without breasts.

*“... I’m gonna do a mastectomy with an immediate reconstruction. I do not want any implants, I don’t want anything foreign in my body. Fortunately I have very small breasts, so they’re able to do it with my own muscle and fat. I really admire those persons who stay flat, because for me I would find it hard to look in the mirror... being young and facing life with this deformity”* (Amy)

A few participants described how they prepared themselves mentally. Maria explained that she looked at images of people who had undergone mastectomies or consulted with friends who had undergone the same experience.

*“So, I was talking to women who did mastectomies, looking up pictures and seeing, ok, so they will have the stitches here and there, they will look flat… so that I prepare myself. I want to be prepared. All the time. That is me. So, looking at pictures, and indoctrinating myself, ok? I will not have any nipples, no areola…”* (Maria)

Despite this, several participants iterated how the surgical removal of their breast tissues affected their confidence across various areas of their lives. Particularly, participants spoke openly about the ramifications upon their sexual wellbeing, stressing how their diminished confidence influenced their interactions during sexual engagement.

*“… even nowadays when I engage sexually, I leave my shirt on, I do not… I do not feel comfortable with my body”* (Alexia)

Following their surgery, participants prepared themselves for the radiotherapy phase.

This involved preparing the skin to minimise burns and peeling:

*“What I did helped me a lot. I used to apply body lotion constantly. I used to apply it everywhere.”* (Christine)

### Nearing the end of my journey

Following the surgery, the participants received adjuvant targeted therapy drugs namely Trastuzumab (Herceptin) and Pertuzumab (Perjeta) for a year. The participants appreciated that this phase of the treatment took up less time. Instead of “taking up 4 hours [for neoadjuvant treatment], this treatment [adjuvant] only takes up an hour, basically half an hour for each drug” (Maria).

While most participants did not encounter significant side-effects during this treatment phase, some reported experiencing fatigue and shortness of breath.

*“I used to experience fatigue some two days after the treatment and shortness of breath, for example if I just climb four steps. I would have to stop as I ended up breathless”* (Christine)

Despite this, the treatment also offered reassurance that any lingering cancer cells would be eliminated, providing participants with a sense of security.

“*When I was told that I was done [from treatment], I took it badly, as I thought Herceptin was my backup preventing any cancer cells from growing and spreading*” (Anne)

“*I processed what I was going through well enough, until I was nearing the end of my treatment [Herceptin]. Because I thought that was the end of the story…it was then that I asked to speak to a counsellor to discuss how I was feeling about the uncertainty of the way ahead”* (Lara)

## Discussion

While prognosis and treatment options vary by subtype of breast cancer, much of the existing research addresses the collective experiences of breast cancer patients. This study contributes to the literature by specifically exploring the lived experiences of non-metastatic women with HER2 + breast cancer, from diagnosis throughout active treatment.

This study underscores, that participants with HER2 + breast cancer placed significant emphasis on the particularly aggressive nature of their condition. Notably, while some participants could not specifically identify their subtype as HER2 + , yet all were acutely aware of the cancer’s aggressiveness and its potential to grow and quickly spread to other organs. This heightened their anxiety both at the time of diagnosis and throughout treatment, due to concerns about metastasis. While previous studies have identified anxiety in breast cancer patients [16,17], there is a dearth in literature relating to whether this outcome varies by subtype of breast cancer.

In response to their uncertainty and anxiety, several participants turned to the internet for information. This is a common practice because of the easy access and the wide availability of up-to-date resources [24]. However, some participants refrained from online searches for various reasons, such as denial of their need for support or encountering conflicting or irrelevant content [25]. This highlights the critical role of health professionals in exploring patient experiences to gain a deeper understanding of their informational needs and concerns, thereby facilitating the inclusion of personalized information in collaboratively developed care plans [26].

Participants further shared that having a relatively common type of cancer with access to targeted, evidence-based treatments, increased their hope for recovery. These treatments enhanced their sense of control over the illness, which is linked to improved vitality and psychological well-being [27]. While participants expressed concerns about treatment side-effects, the perceived necessity of treatment created a sense of urgency to begin and adhere to it. Many took extensive precautions, such as avoiding contact with individuals who were ill, to ensure that they did not miss treatments. These findings align with the Necessity-Concerns framework, which posits that adherence to treatment is stronger when beliefs about its necessity outweigh concerns [28].

As participants approached the end of their treatment, they reported feeling vulnerable, fearing the loss of a vital support system and the possibility of relapse. This concurs with Williams and Jeanetta’s findings [16], which indicate that for breast cancer patients, the recovery process often feels incomplete on the completion of medical treatment. This insight highlights the need for ongoing support post-treatment to address persistent fears and navigate the transition period following treatment completion.

Overall, participants demonstrated a “fighting spirit,” perceiving their treatment as a battle against cancer. Their determination to overcome this aggressive form of cancer was largely driven by a desire to remain present for their families, particularly for their children. Similar sentiments have been reported in other studies involving persons diagnosed with breast cancer [17,29,30]. Additionally, participants efforts to regain control and adapt to their circumstances following a breast cancer diagnosis often led to an opportunity to redefine themselves and discover meaning in what might otherwise feel like a chaotic and purposeless existence [31,32]. This aligns with the concept of post-traumatic growth, which encompasses finding purpose in life, strengthening relationships, developing personal resilience, exploring new opportunities, and nurturing spirituality [33,34].

Participants also discussed the physical changes that affected their female identity. They grappled emotionally with embracing their “new” bodies, which diverged from socially accepted norms, and expressed concerns about the impact on their families. These findings concur with those of Ciria-Suarez et al. [29] and Grogan et al. [35], where participants also struggled to accept their bodies after cancer and used strategies to conceal their cancer-altered bodies from others. Additionally, participants expressed concerns about how their diagnosis might impact their ability to fulfil their maternal responsibilities. This was also highlighted in the systematic review by Kuswanto et al. [30] who further explored the coping strategies used by mothers to adjust to their changing roles in parenting.

The social implications of a breast cancer diagnosis were also evident, with support provided by a small circle of family and friends. This may occur because individuals with cancer view their condition as a private matter and/or because those providing support may struggle to understand the complexities of living with cancer, potentially straining relationships [29,36].

In parallel, participants expressed mixed emotions about the type of surgery they should undergo. For some, a mastectomy was perceived as necessary to reduce the risk of recurrence, while others prioritised body image concerns in their decision-making process. This underscores the importance of healthcare professionals understanding patients’ beliefs and values and discussing options to include personalized information in care plans [37], even when the primary focus is on extending life expectancy [38].

Participants also described striving to identify potential triggers for their cancer. This allowed them to exert some control over their situation and find meaning in their illness [39]. Some attributed their condition to genetics and were distressed that their children might face a similar fate (i.e., a HER2 + diagnosis). Others reflected on their lifestyle, questioning whether it was the cause of their illness, and why individuals with less healthy lifestyles did not share the same outcome. Although maintaining a healthy lifestyle is associated with a decreased risk for breast cancer [40,41], it is important to educate the public that other factors such as genes, reproductive factors and age may also predispose persons to a cancer diagnosis [42].

### Implications for practice

Despite medical advances in treating HER2 + breast cancer, this study underscores the need for continuous support for patients throughout the diagnostic, active treatment and post treatment phase. Health professionals play a crucial role in providing person-centred care by exploring patient narratives to understand their unique concerns and needs. This approach enables personalised support that focuses on individual experiences, coping styles, and changing life situations throughout treatment.

The study also highlights the participants’ intense focus on the cancer’s aggressiveness and fear of metastasis. Professionals need to create a context where persons with breast cancer feel at ease to discuss their concerns and also seek information. Additionally, cognitive behavioral therapy can be utilised to reshape negative thoughts and reduce mental health consequences.

Support requirements must also be addressed as treatment is nearing the end. Healthcare professionals should discuss patients’ fears about a recurrence when treatment stops and their anxiety about where to find support. The transition away from healthcare services after completing treatment should be gradual, allowing individuals time to adjust to a more independent phase while ensuring they have the necessary information to access support.

### Strengths and limitations

To the knowledge of the present authors, this study is the first of its kind to explore the lived experiences (i.e., from diagnosis throughout the treatment trajectory) specifically of women diagnosed with non-metastatic HER2 + breast cancer. As typical of IPA research however, this study was conducted on a small sample of female participants, hence, the findings cannot be generalised to a wider population. However, the aim of the study was not to generalise the findings, but rather to focus specifically on the sample under investigation.

## Conclusion

This study reveals the multifaceted impact of HER2 + breast cancer on women. Findings reveal specific insight into the experiences of women throughout the various stages of the cancer trajectory, with much of the participants’ concerns surrounding the aggressiveness of this specific breast cancer subtype. It is important for healthcare professionals to gain insight into their concerns and needs, to be in a better position to provide care that centres around their unique demands. The need for enhanced education about risk factors of breast cancer and information targeting survivorship was highlighted, which can help individuals make informed choices. Support services must be developed and implemented as part of the treatment programme, enabling an acceptance of the diagnosis and the development of effective coping strategies.

## References

[1] Bray F, Ferlay J, Soerjomataram I, Siegel RL, Torre LA, Jemal A. Global cancer statistics 2018: GLOBOCAN estimates of incidence and mortality worldwide for 36 cancers in 185 countries. CA Cancer J Clin. 2018;68(6):394–424. doi: 10.3322/caac.21492 30207593

[2] Global Cancer Observatory. Breast cancer statistics. https://www.wcrf.org/cancer-trends/worldwide-cancer-data/. 2022. Accessed 2023 October 27.

[3] Asif HM, Sultana S, Ahmed S, Akhtar N, Tariq M. HER-2 Positive Breast Cancer - a Mini-Review. Asian Pac J Cancer Prev. 2016;17(4):1609–15. doi: 10.7314/apjcp.2016.17.4.1609 27221828

[4] Wang J, Xu B. Targeted therapeutic options and future perspectives for HER2-positive breast cancer. Signal Transduct Target Ther. 2019;4:34. doi: 10.1038/s41392-019-0069-2 31637013 PMC6799843

[5] De Mattos-Arruda L, Cortes J. Advances in first-line treatment for patients with HER-2+ metastatic breast cancer. Oncologist. 2012;17(5):631–44. doi: 10.1634/theoncologist.2011-0187 22523199 PMC3360903

[6] Akhavan A, Binesh F, Hoseini-Dezki SS, Mortazavizadeh SMR. Evaluation of the Survival of HER2-Positive Breast Cancer Patients Receiving Herceptin Compared With Those Who Did Not Receive It. IMMINV. 2018;3(2):63. doi: 10.24200/imminv.v2i4.122

[7] Pegram M, Pietras R, Dang CT, Murthy R, Bachelot T, Janni W, et al. Evolving perspectives on the treatment of HR+/HER2+ metastatic breast cancer. Ther Adv Med Oncol. 2023;15:17588359231187201. doi: 10.1177/17588359231187201 37576607 PMC10422890

[8] Harbeck N, Burstein HJ, Hurvitz SA, Johnston S, Vidal GA. A look at current and potential treatment approaches for hormone receptor-positive, HER2-negative early breast cancer. Cancer. 2022;128 Suppl 11:2209–23. doi: 10.1002/cncr.34161 35536015

[9] Choong GM, Cullen GD, O’Sullivan CC. Evolving standards of care and new challenges in the management of HER2-positive breast cancer. CA Cancer J Clin. 2020;70(5):355–74. doi: 10.3322/caac.21634 32813307

[10] He X, Yeung S-CJ, Esteva FJ. A new paradigm for classifying and treating HER2-positive breast cancer. Cancer Rep (Hoboken). 2023;6(8):e1841. doi: 10.1002/cnr2.1841 37254964 PMC10432420

[11] Cardoso F, Paluch-Shimon S, Senkus E, Curigliano G, Asapro M, Andre F, et al.5th ESO-ESMO international consensus guidelines for advanced breast cancer (ABC 5). Ann Oncol. 2020;31(12):1623–49. doi: 10.1016/j.annonc.2020.09.010 32979513 PMC7510449

[12] Pegram M, Jackisch C, Johnston SRD. Estrogen/HER2 receptor crosstalk in breast cancer: combination therapies to improve outcomes for patients with hormone receptor-positive/HER2-positive breast cancer. NPJ Breast Cancer. 2023;9(1):45. doi: 10.1038/s41523-023-00533-2 37258523 PMC10232442

[13] Burstein HJ. Adjuvant systemic therapy for HER2-positive breast cancer. https://www.uptodate.com/contents/adjuvant-systemic-therapy-for-her2-positive-breast-cancer. 2023. Accessed 2023 November 13.

[14] Martinez-Saez O, Prat A. Current and future management of HER2-positive metastatic breast cancer. American Soc Clinical Oncol. 2021;17(10). doi: 10.1200/DP.21.00172 34077236

[15] Lewis S, Yee J, Kilbreath S, Willis K. A qualitative study of women’s experiences of healthcare, treatment and support for metastatic breast cancer. Breast. 2015;24(3):242–7. doi: 10.1016/j.breast.2015.02.025 25753212

[16] Williams F, Jeanetta SC. Lived experiences of breast cancer survivors after diagnosis, treatment and beyond: qualitative study. Health Expect. 2016;19(3):631–42. doi: 10.1111/hex.12372 25953316 PMC5029767

[17] Kim J, Lee K. Lived experiences of breast cancer in patients under the age of 40: A phenomenological study. Eur J Oncol Nurs. 2023;65:102336. doi: 10.1016/j.ejon.2023.102336 37339554

[18] Novitarum L, Siregar MFG, Siregar FA, Lubis NL. Survivor’s experience in fighting breast cancer. J Popul Ther Clin Pharmacol. 2022;29(4):e126–33. doi: 10.47750/jptcp.2022.976 36441050

[19] Galipeau N, Klooster B, Krohe M, Tang DH, Revicki DA, Cella D. Understanding key symptoms, side effects, and impacts of HR+/HER2- advanced breast cancer: qualitative study findings. J Patient Rep Outcomes. 2019;3(1):10. doi: 10.1186/s41687-019-0098-1 30734110 PMC6367496

[20] Siddiqi A, Given CW, Given B, Sikorskii A. Quality of life among patients with primary, metastatic and recurrent cancer. Eur J Cancer Care. 2009;18(1):84–96. doi: 10.1111/j.1365-2354.2008.01021.x 19484831

[21] Smith JA, Larkin M, Flowers P. Interpretative Phenomenological Analysis: Theory, Method and Research. London, UK: Sage. 2009.

[22] Larkin M, Watts S, Clifton E. Giving voice and making sense in interpretative phenomenological analysis. Qual Res Psychol. 2006;3(2):102–20. doi: 10.1191/1478088706qp062oa

[23] Yardley L. Dilemmas in qualitative health research. Psychol Health. 2000;15(2):215–28. doi: 10.1080/08870440008400302

[24] Cotten SR, Gupta SS. Characteristics of online and offline health information seekers and factors that discriminate between them. Soc Sci Med. 2004;59(9):1795–806. doi: 10.1016/j.socscimed.2004.02.020 15312915

[25] Lazard AJ, Collins MKR, Hedrick A, Horrell LN, Varma T, Love B, et al. Initiation and changes in use of social media for peer support among young adult cancer patients and survivors. Psycho-Oncol. 2021;30(11):1859–65. doi: 10.1002/pon.5758 34165848

[26] Banks D, Scerri J, Davidson J. Being person-centred in mental health services. In: McCormack B, McCance T, Bulley C, Brown D, McMillan A, Martin S, editors. Fundamentals of Person-Centred Healthcare Practice. Oxford: Wiley Blackwell. 2021. p. 209–18.

[27] Hagger MS, Orbell S. A Meta-Analytic Review of the Common-Sense Model of Illness Representations. Psychol Health. 2003;18(2):141–84. doi: 10.1080/088704403100081321

[28] Phillips LA, Diefenbach MA, Kronish IM, Negron RM, Horowitz CR. The necessity-concerns framework: a multidimensional theory benefits from multidimensional analysis. Ann Behav Med. 2014;48(1):7–16. doi: 10.1007/s12160-013-9579-2 24500078 PMC4104213

[29] Ciria-Suarez L, Jiménez-Fonseca P, Palacín-Lois M, Antoñanzas-Basa M, Fernández-Montes A, Manzano-Fernández A, et al. Breast cancer patient experiences through a journey map: A qualitative study. PLoS One. 2021;16(9):e0257680. doi: 10.1371/journal.pone.0257680 34550996 PMC8457460

[30] Kuswanto CN, Stafford L, Sharp J, Schofield P. Psychological distress, role, and identity changes in mothers following a diagnosis of cancer: A systematic review. Psycho-Oncol. 2018;27(12):2700–8. doi: 10.1002/pon.4904 30289196

[31] Loeffler S, Poehlmann K, Hornemann B. Finding meaning in suffering?-Meaning making and psychological adjustment over the course of a breast cancer disease. Eur J Cancer Care (Engl). 2018;27(3):e12841. doi: 10.1111/ecc.12841 29575157

[32] Magnus E, Jakobsen K, Reidunsdatter RJ. Meaningful everyday life projects and activities in breast cancer survivors. Moravian Geographical Rep. 2020;28(4):299–307. doi: 10.2478/mgr-2020-0022

[33] Lindberg P, Koller M, Steinger B, Lorenz W, Wyatt JC, Inwald EC, et al. Breast cancer survivors’ recollection of their illness and therapy seven years after enrolment into a randomised controlled clinical trial. BMC Cancer. 2015;15(1). doi: 10.1186/s12885-015-1573-6 PMC4517565 26219863

[34] Galea M, Scerri J, Grech P, Sammut A, Attard C. Post-Traumatic Growth after Cancer: A Thematic Analysis Study. HSSR. 2023;6(1):p11. doi: 10.30560/hssr.v6n1p11

[35] Grogan S, Mechan J, Persson S, Finlay S, Hall M. “I’ve got a very dichotomous difference in the way that I perceive myself”: positive and negative constructions of body image following cancer treatment. J Health Psychol. 2019;24(10):1368–77. doi: 10.1177/1359105317730896 28929823

[36] Fong AJ, Scarapicchia TMF, McDonough MH, Wrosch C, Sabiston CM. Changes in social support predict emotional well-being in breast cancer survivors. Psychooncology. 2017;26(5):664–71. doi: 10.1002/pon.4064 26818101

[37] McCance T, McCormack B. The person-centred framework. In: McCormack B, McCance T, editors. Person-Centred Practice in Nursing and Health Care. Oxford: Wiley Blackwell. 2017. p. 36–64.

[38] Lyons-Rahilly T, Meskell P, Carey E, Meade E, O’ Sullivan D, Coffey A. Exploring the experiences of women living with metastatic breast cancer [MBC]: A systematic review of qualitative evidence. PLoS One. 2024;19(1):e0296384. doi: 10.1371/journal.pone.0296384 38181009 PMC10769043

[39] Janoff‐Bulman R, Frieze IH. A Theoretical Perspective for Understanding Reactions to Victimization. J Soc Issues. 1983;39(2):1–17. doi: 10.1111/j.1540-4560.1983.tb00138.x

[40] El Sharif N, Khatib I. Healthy Lifestyle and Breast Cancer Risk in Palestinian Women: A Case-Control Study. Nutr Cancer. 2023;75(3):901–11. doi: 10.1080/01635581.2023.2168022 36655429

[41] Khalis M, Chajès V, Moskal A, Biessy C, Huybrechts I, Rinaldi S, et al. Healthy lifestyle and breast cancer risk: A case-control study in Morocco. Cancer Epidemiol. 2019;58:160–6. doi: 10.1016/j.canep.2018.12.012 30597481

[42] Sun Y-S, Zhao Z, Yang Z-N, Xu F, Lu H-J, Zhu Z-Y, et al. Risk factors and preventions of breast cancer. Int J Biol Sci. 2017;13(11):1387–97. doi: 10.7150/ijbs.21635 29209143 PMC5715522

